# 

**Published:** 1957-10

**Authors:** 


					THE
MEDICAL JOURNAL
OF THE
SOUTH-WEST
Incorporating
The Bristol Medico-Chirurgical Journal
October 1957
72 (iv) N0> 266 Price 4/-

				

